# An Unknown Renaissance Portrait of Tagliacozzi (1545–1599), the Founder of Plastic Surgery

**DOI:** 10.1097/GOX.0000000000002006

**Published:** 2019-01-04

**Authors:** Sophie Ménard

**Affiliations:** From Yale University, New Haven, Conn.

## Abstract

This article examines a recently discovered painting of a young scholar holding a reconstructed nose. Experts in ancient paintings have confirmed that the portrait is an authentic painting of the School of Bologna in Italy, from the last quarter of the Renaissance. In the 1580s, Gaspare Tagliacozzi (1545–1599), a young professor in surgery and anatomy at the University of Bologna, Italy, was the only one to carry out reconstructions of the nose and other missing parts of the face. We have looked whether different relevant components of this painting, which is presented for the first time to the medical community, could match with Tagliacozzi’s life and achievements. We have also compared the portrait to another portrait of Tagliacozzi painted circa 1597, which belongs to the institute Rizzoli in Bologna, Italy. The latter depicts Tagliacozzi as an older established surgeon. He is seen presenting his illustrated book, *De Curtorum Chirurgia per Insitionem (On the surgical restoration of defects*), which is the first book devoted to plastic surgery. We have concluded that the young Renaissance scholar is Tagliacozzi. This portrait and the Rizzoli’s portrait represent Tagliacozzi at the beginning and at the peak of his professional involvement in the field of plastic surgery. Tagliacozzi is the first medical doctor to practice plastic surgery as well as write about it. He also taught plastic surgery for the first time in a prestigious Renaissance school of medicine. Tagliacozzi’s illustrated book of plastic surgery published in 1597, disrupted the contemporary medical community. For all these reasons, Tagliacozzi can be considered as the founder of plastic surgery. Unfortunately, he died at the age of 54, which put a term to the development of this field. Tagliacozzi’s work was rediscovered, 2 centuries later, by the English surgeon J.C. Carpue (1764–1846) during the revival of plastic surgery.

## INTRODUCTION

In the Renaissance, mutilations of the face and particularly of the nose, were common as a result of years of endemic wars, syphilis, duels, and corporeal punishments. The victims who suffered from mutilated noses were mostly doomed for the rest of their lives and had to rely on clever artifices to mask their deformities. They often wore prosthetic noses, made of silver, gold, or even leather, which were then attached to glasses or tied around their heads.

In the 15th century, most operations were done by barber-surgeons who did not depend on the academia, did not have any medical degrees and knew neither Greek nor Latin. Despite their lack of university education, they were resourceful and talented when it came to facial reconstructions. The art of restoring nose mutilation with a skin flap taken from the arm (or the Italian method) was first attempted by the Sicilian barber-surgeon Branca.^1^

## DISCUSSION

### The Italian Method for Nose Reconstruction

Branca carried out a method to reconstruct noses using a skin flap from the arm, but he kept his technique secret. His method consisted of the following: a piece of skin (or a flap) from the arm, was cut in the shape of the missing nose. The proximal part of the flap was then lifted from the patient’s arm. After a period of time, the detached part of the flap was sutured to the nasal stump. A splint was used to immobilize the patient’s arm by linking it to his face. The flap was relieved a few weeks after, liberating the arm. After a final period of time, the rest of the flap was folded to form the nasal tip and stitched to the face. The German barber-surgeon, Pfoslpeundt, probably knew the method by word of mouth from this Italian barber-surgeon and successfully carried out a similar operation in 1460.^2^

The method for nose reconstruction was briefly described for the first time in a book of anatomy by A.Benedetti (1460–1512) published in Latin in 1502 Venice, Italy. Posthumous editions of his *Anatomicae* were published in 1514 Paris, France,^3^ and in 1527 Cologne, Germany. However, the exact description of the nose reconstruction in a widespread remarkable textbook of anatomy did not seem to spark any interest at the time for the medical community.

In the 16th century, the Italian method was taken over by the Vineos, a barber-surgeon family from Calabria, Italy.^4^

The brilliant G. Fallopius (1523–1562) was against these operations and wrote that he “would rather lack a nose than undergo this treatment” and that he would “advise the use of an artificial nose rather undergo such a torture.”^5^ The Italian method for nose reconstruction was reported in A. Vesale (1514–1564) and P. Borgarucci’s (?-?) book *Chirurgia Magna*, published in Latin in 1568.^6^ But the authors described wrongly the technique, indicating that a part of the biceps muscle was used to reconstruct the nose. The renowned French surgeon A. Paré (1510–1590) repeated the same mistake in his *Oeuvres*, published in French in 1575.^7^ This might be the reason for why so many medical doctors criticized nose reconstructions because of its complexity and because of the pain caused by using part of the biceps muscle.

**Fig. 1.**
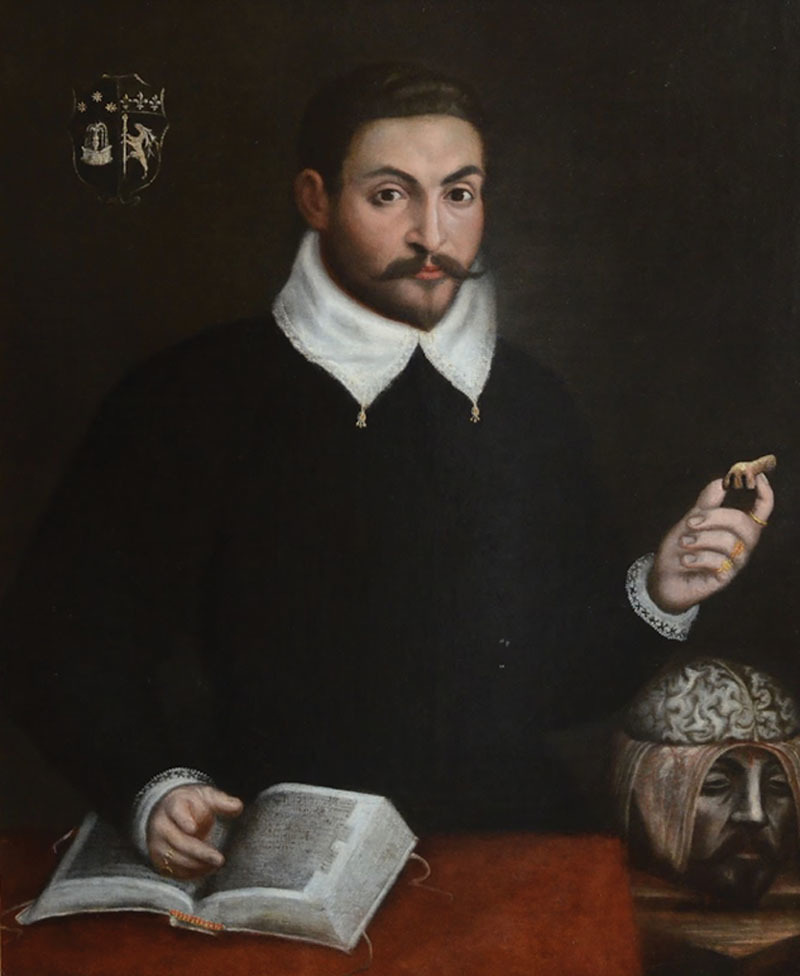
Portrait of Tagliacozzi young. Circa 1580. Full view of the painting.

**Fig. 2.**
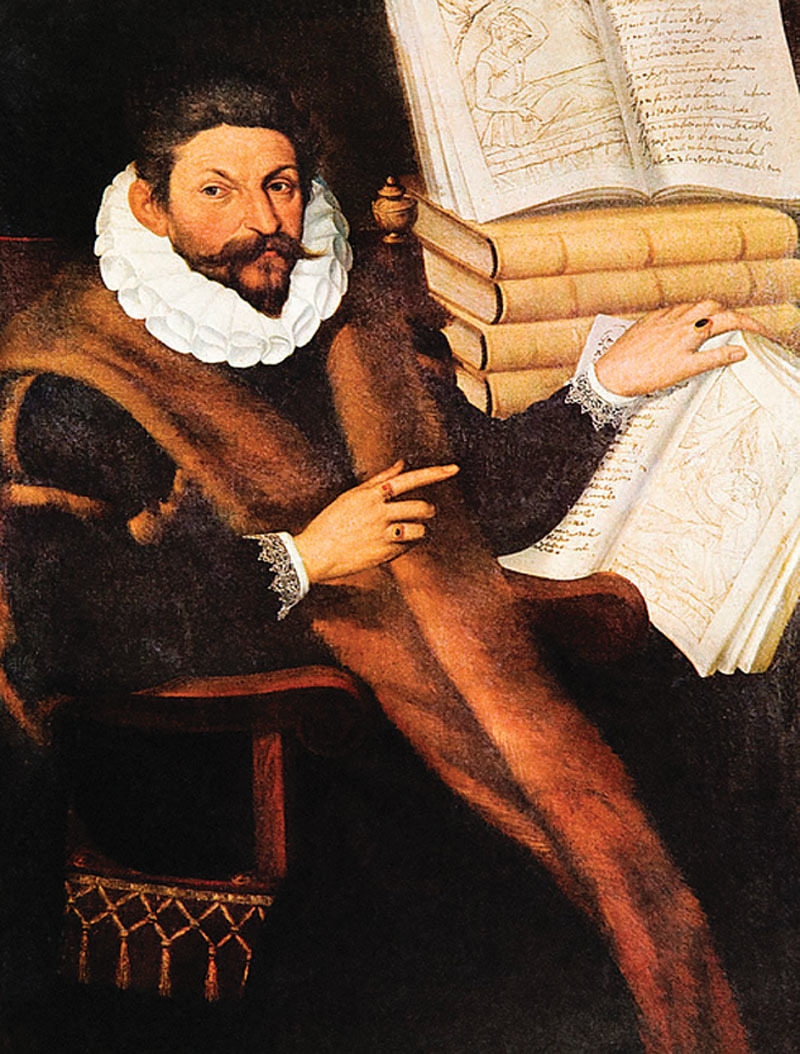
Rizzoli portrait of Tagliacozzi. Circa 1597. Full view of the painting.

Despite these barbers’ secret traditions, wrong descriptions in contemporary surgical textbooks and often poorly executed operations, one might wonder how the practice of nose reconstructions or other parts of the face finally became accepted in the last quarter of the Renaissance by the medical and scholarly communities. After the recent discovery of a portrait depicting a young Italian Renaissance scholar holding an entirely reconstructed nose (Fig. 1), we researched the origins of plastic surgery, which were exclusively located in the end of the Renaissance, at the University of Bologna, Italy. We found evidence for Gaspare Tagliacozzi’s (1545–1599) pivotal role as an academic in this new field. We looked whether different relevant components of the painting were linked to Tagliacozzi’s beginning career, since there are no other known paintings of him as a young surgeon.^8,9^ We also compared the portrait to the Rizzoli’s portrait of Tagliacozzi painted 2 years before his death (Fig. 2)^10^ and came to the conclusion that this young scholar is that of none other but the pioneer of plastic surgery.

### The Portrait of a Young Italian Renaissance Scholar Holding a Reconstruct Nose

This is the first time the painting is presented to the medical community (Fig. 1). The oil on canvas is 95.5 cm in height and 73.5 cm in width. The painting has been identified by the Cabinet Turquin in Paris, which specializes in ancient paintings.^11^ According to the expert, this oil on canvas was painted toward the last quarter of the 16th century and bears characteristics of the Bolognese school of painting. Toward the end of the Renaissance era, this type of portrait was in high demand. It was an opportunity for scholars to promote themselves as well as their fields of study. They were represented in their professional attire, sat in front of an office, were turned toward the viewer and pointed out the objects of their study. In a way, they served the purpose of curriculum vitae or Linkedin profiles. These paintings were expensive and their price depended on the notoriety of the painter.

In this painting (Fig. 1), the young scholar has a very serious gaze. He is sitting up straight and confident. He is wearing a black scholarly robe with a white, finely embroidered collar. His face is framed by bushy, arched eyebrows and dark lush hair. There is not a single wrinkle on his forehead. His lids have well-defined folds. His right ear is protruding. His face is quite long, with a well-trimmed beard and mostache. One can also notice the small bump on his long nose.

The representation of the dissected head meant that he was allowed to do anatomical dissections. It also highlights the striking contrast between the living reconstructed nose he holds and the blood-emptied nose of the dissected head (Fig. 1). This suggests that the scholar did not only practice dissection but also surgical reconstructions of the nose. The anatomical head could be a specific reference to the University of Bologna in the field of neuro-anatomy with famous alumni like Mundino de Luzzi (1270–1326) and J. Berengario da Carpi (1457–1530). It could also refer to the recent groundbreaking discoveries of JC. Arentius (1530–1589) and C. Varolius (1543–1575), who were both professors at the University of Bologna. Arentius had discovered the hippocampus, also known as the memory’s kingdom. Varolius had discovered the bridge that links the 2 hemispheres of the brain.

In the portrait (Fig. 1), the right hand of the scholar rests on an open book (which could very well be his manuscript) without illustrations. The whiteness of the paper, the darkness of the black ink, the vellum book binding with its undamaged strings ties, and the lightness of the colored upper head banding, all seem to suggest that the book or the manuscript is recent.

Lastly, a crucial element of the painting is the coat of arms, which is located on the upper left (Fig. 1). One can notice a fountain, a symbol that serves to identify the family Tagliacozzi blazon.^12,13^

### Tagliacozzi’s Medical Career at the University of Bologna: the birthplace of plastic surgery

Tagliacozzi came from a Bolognese silk-weaver family. In 1565, when he was 20 years old, he began his 5-year long medical school at the University of Bologna, Italy. One of his teachers was J.C. Arentius, a professor of surgery and anatomy who conducted both surgical operations and anatomical dissections.

The first event that could be tied back to Tagliacozzi and plastic surgery during his medical studies is the following. Oczko (1537–1599), a Polish doctor, visited the University of Bologna between 1565 and 1569. He reported on the topic of nose reconstruction in his *Treaty on Syphilis*, Krakow, Poland (1581).^14^ He commented Vesale’s method using the biceps muscle and wrote “believe me, this is not an easy thing to do.” Then, Oczko wrote that he witnessed Arentius during his stay in Bologna carrying out a nose reconstruction “using the skin of the arm which is much easier.”^15^

Oczko is the only one who reported such an extraordinary operation even though many scholars visited the University of Bologna in search of knowledge. This multi-stage operation was probably carried out by Arentius just before Oczko left Bologna, in 1569. Oczko did not see the final result because he concluded his description of the Italian method using the conditional “the arm would not be much harmed and a good looking nose would be reconstructed.”^15^ Arentius did not mention any nose reconstructions in his own books, which could suggest that his operation did not end successfully.

The second event that could be related to Tagliacozzi and plastic surgery is the first edition in Italian in 1570 by L. Fioravanti (1517–1588) of the *Tesoro della vita humana*.^16^ Fioravanti, a Bolognese barber-surgeon, was in Bologna between 1568 and 1570.^17^ He was hoping that his numerous publications and connections would help him get a medical degree from this prestigious university. Fioravanti described in his book the secret Italian method for nose reconstruction. In chapter 27 of the second book, he wrote “when the surgery started, I pretended, by trickery, that I had not the courage to see such a sight and turned my face away, yet my eyes saw perfectly the whole secret.”^18^

Fioravanti also wrote that the operation was carried out by the 2 brothers, Pietro and Paolo Vineo, barber-surgeons from Calabria, Italy. However, only the last Vineo, Bartolomeo was still living and doing nose reconstruction circa 1570.^18^ That means Fioravanti reported a nose reconstruction that took place much earlier.

In all his previous treaties, Fioravanti had never described the Italian method. In 1555, he had only mentioned “in the kingdom of Naples there are also men who remake noses for those who have lost them” but he did not give additional details on the operations they did.^19^ Fioravanti’s decision to write for the first time a chapter on nose reconstruction in 1570 was actually not accidental.

In fact, Fioravanti may have implicitly given a correction to the erroneous description of nose reconstruction in the Vesale and Borgarucci’s Chirurgia Magna, published in 1568.^6^ Maybe it was Fioravanti’s way to teach a lesson to the Bolognese medical community and particularly to Arentius after his unsuccessful nose reconstruction. Clearly, he felt superior and confident in his skills when writing “I learnt the nose reconstruction as well as the Vineos and thus should I wish I could do it …”^18^ In the end, Fioravanti did not succeed in getting his medical degree from the University of Bologna^19^ even if he claimed he did.^17^

All these previous accounts prove that nose reconstructions were carried out in the University of Bologna—but it is hard to determine whether or not they ended up being successful, because of the secrecy and jealousy within the medical community.

After his graduation from medical school in 1570, Tagliacozzi was appointed professor of surgery at the University of Bologna. In 1576, he also graduated in philosophy and was admitted to the doctoral college of Medicine and Philosophy. Later in 1576, Tagliacozzi started his private practice. In January 1579, Cardinal A. Sforza (1534–1581) signed a decree from the Senate of Bologna,^20^ allowing Tagliacozzi to carry out anatomical dissections. This crucial information allows us to conclude that the portrait (Fig. 1) could have been painted in the early 1580s, when Tagliacozzi was approximately 35 years old, at the start of his promising career after obtaining the authorization for anatomical dissections.

The following inscription, engraved in Latin on a marble table from January 16th, 1582, tells us about Tagliacozzi rising fame. Here is the translation given by JC. Carpue^21^: “To the most learned and most excellent man, Gaspare Tagliacozzi. We celebrate your genius, worth and skill; both for the scientific dissections performed by your hand and still more for the unseen benefits which you hast heaped upon us and which the tombs of men conceal. Therefore for yours merits live forever in this marble you man of genius, worth, science and skill!”. We know that Tagliacozzi had been noticed by several distinguished doctors. Nose reconstructions carried out by Tagliacozzi were reported for the first time in 1583 by the German doctor M. Holtzapfel (?-?).^22^ He also mentioned Tagliacozzi’s fame and reputation throughout Italy. G. Mercuriale (1530–1606), a Renaissance star professor, head of the medicine department at the University of Padua, Italy, wittily reported in his De Decoratione Liber^23^: “the excellent Tagliacozzi recently showed me, while I was on a visit in Bologna, 2 patients whose noses he has restored. Of course the article (the reconstructed nose) is not so completely similar that no discrepancy can be detected but it is really a great advance.”

A feud between Arentius and Tagliacozzi arose in 1584. The good relationship between Arentius and Tagliacozzi, who were both professors of surgery and anatomy at the same university, changed abruptly. Dissections of criminals’ bodies donated to a medicine school were strictly regulated at the time, and were generally allowed only once a year. A scandal occurred during a lesson on anatomy involving a dissection in February 1584. Tagliacozzi students could not perform the dissection because his designated subject to be dissected had been snatched by Arentius without any explanations.^24^ This quarrel spread throughout the university and even reached the doors of the senate of Bologna …

Now, what could explain such a rivalry between the two professors who had worked side by side for years? This event could have been triggered by Tagliacozzi rising fame and eminence in the field of plastic surgery. Was Arentius resentful towards his former student’s success? Was he bitter because Tagliacozzi had failed to acknowledge him, his mentor, an established surgeon who had already carried out a nose reconstruction more than a decade before Tagliacozzi did his own?

On February 22, 1586, Tagliacozzi shared the first complete description of his own experience with nose reconstruction in Mercuriale second edition *De Decoratione Liber*, which was published in 1587 in Frankfurt, Germany.^25^ Tagliacozzi also informed Mercuriale that he had almost finished his treaty on plastic surgery. He explained that the publication of the book had to be postponed due to a lack of illustrations. This important detail takes us back to the painting we present, with the open book or manuscript. In the painting, there are no illustrations on the pages of the open book (Fig. 1).

During the following years, Tagliacozzi continued his practice of plastic surgery. T.Feyens (1567–1631), a native of Antwerp, Holland went in 1590 to Bologna and became a student of Tagliacozzi. He wrote about Tagliacozzi that “he restored a large number of noses some of which I saw restored upon their owners others in process of being built up.”^26^

F.von Hilden (1560–1634) reported a case of J. Griffon^27^ who reconstructed the nose of woman in Lausanne, Switzerland, in 1592, using Tagliacozzi’s technique which he had learned by word of mouth from one of Tagliacozzi’s patients. F. Licetis (1577–1656) wrote in 1595: “we have often seen Tagliacozzi restoring mutilated parts of the face “^28^ It seems like Tagliacozzi’s skills made him a unique reference in Europe for plastic surgery. All these testimonials prove that Tagliacozzi was a star in his field, and foreign doctors or students were lucky to assist to his operations and witness his talents in plastic and reconstructive surgery.

### The Rizzoli Portrait of Tagliacozzi^10^

In the Rizzoli portrait, Tagliacozzi is a mature, successful, and wealthy professor. He looks very satisfied, his posture is less straight compared with our portrait. Tagliacozzi sat in a luxury armchair. He is wearing a splendid and expensive black scholarly robe with fur, a long stole in fur, very finely embroidered cuffs and a beautiful ruff. His hands are decked with precious rings. His face resembles strikingly the first portrait but older, his eyebrows are not as full and his hair is more grayish. His lids have less well defined folds, and one can notice his under-eye bags. This right nasolabial fold is sagging. His right ear is protruding. His face is quite long, with a very similar beard and mostache. His nose is long with a bump and a falling tip. Tagliacozzi is clearly pointing to the illustration of his prominent book *De Curtorum Chirurgia per Insitionem (on the surgical restoration of defects*). Two copies are seen open, with illustrations of the technique of nose reconstruction. The trick of representing many copies opened or unopened of the same book could have been done one purpose to underline the importance of the work. Even though this portrait was not signed, it was probably from the hand of Tiburzio Passarotti (1553–1612), a renowned Bolognese painter who was praised by Tagliacozzi.^29^

### Tagliacozzi’s Plastic Surgery Book

Tagliacozzi’s plastic surgery in folio book *De Curtorum Chirurgia per Insitionem* was first published in 1597 by Bindoni, in Venice, Italy.^30^ In the preface, Tagliacozzi explained that “very little is left to us by ancient writers on the supplying of deficient part of the body. The restoration of lips, ears and noses, is said to have been formerly practiced in Calabria, but the art… was rather pursued at random than according to any fixed method. On my part… I have placed this particular branch upon the basis of uniform rules and have reduced the method of practice into writing.” Throughout his book Tagliacozzi expressed his forward-thinking very modern motto: “restore, rebuild and make whole those parts which nature has given but which fortune has taken away. Not so much it may delight the eye, but that it might buoy up the spirit and help the mind of the afflicted.”^30^

In his book on the history of medical illustrations, Herrlinger wrote about Tagliacozzi’s work that “The twenty-two illustrated plates represent for the first time an atlas of plastic surgery and reveal considerable artistic skill. They mark a turning point in Renaissance surgical illustration. The illustrations serve the purpose of didactic representation, which means that the reader could clearly learn from them - and if even emulate the operation. For the first time, the arrangement of a surgical operation is well thought out and presented step by step: the most important instruments, the first three stages of the operation, the plates showing the bandages, and finally the illustrations showing the removal of the bandage and the separation of the skin flap. Other plates illustrate the operation for shaping the nostrils, last plates illustrate the plastic surgery of lips and ears by means of simple line-drawings.”^31^

In the same year, a pirated smaller folio edition with a shorter text and simpler illustrations was published in Venice, Italy by R. Meietti^30^ as a result of the successful demand for the book in the medical community. A third, in octavo, illustrated edition with a different title was published in Frankfurt, Germany in 1598.^32^ However, after 2 years of great interest, Tagliacozzi’s work fell into oblivion soon after his death in 1599.

## CONCLUSIONS

These 2 Renaissance paintings sum up well the professional life and achievements of Tagliacozzi. He is represented at the beginning and at the peak of his successful career as the founder of plastic surgery.

Tagliacozzi’ portraits depict how he worked endlessly to develop plastic surgery, spread his knowledge and transmit his practice. As a young scholar, Tagliacozzi wanted to emphasize that the primary tools for a practitioner, are his hands which he uses for anatomical dissection and surgery. Tagliacozzi also makes the claim that nose reconstruction is in the hands of an academic surgeon and anatomist who shares his knowledge with medical students, visitors, and the entire global medical community of the Renaissance. The second crucial tool is the book, in the 2 paintings, which transmits the knowledge and savoir-faire.

Unfortunately, Tagliacozzi died in 1599 when he was only 54 years old and this halted the blossom of plastic surgery. After Tagliacozzi’s death, the Catholic Church, which shunned his operations, deeming that they meddled with the work of God, is said to have exhumed his body and reburied it in unconsecrated ground. Throughout the next 2 centuries Tagliacozzi’s principles of plastic surgery was mostly ignored, denigrated, or mocked by surgeons and never practiced in Europe. It was not until 1816 that J.C. Carpue, a member of the Royal College of surgeon of London, England, brought back Tagliacozzi’s work into the light. Carpue’s book^21^ was translated in 1817 in German^33^ with a foreword of CF. von Graefe, a professor of surgery in Berlin, Germany. In 1817, Graefe performed for the first time in centuries the Tagliacozzi’s method of nose reconstruction.^34^ This operation triggered the revival and development of plastic surgery for the following 2 centuries.

## ACKNOWLEDGMENTS

The authors thank Mr. Pinta and Mr. Turquin for their expertise on the painting. The authors would also like to thank professors Millicent Marcus, Shiri Goren, and Peter Cole.
